# Aggressive digital papillary adenocarcinoma diagnosed by fine needle aspiration cytology

**DOI:** 10.4103/0970-9371.71878

**Published:** 2010-07

**Authors:** Jayashree Krishnamurthy, Bindu Patil

**Affiliations:** Department of Pathology, Medical College (VIMS), Bellary - 583 104, Karnataka, India

**Keywords:** Aggressive digital papillary adenocarcinoma, eccrine sweat gland tumor, fine needle aspiration cytology

## Abstract

Aggressive digital papillary adenocarcinoma is a rare variant of eccrine sweat gland malignancy with a propensity for metastases and recurrence. We report a 45-year-old female with aggressive digital papillary adenocarcinoma diagnosed by fine needle aspiration cytology (FNAC). The cytological findings were representative of the histological features. The recognition of aggressive digital papillary adenocarcinoma as a distinct clinicopathological eccrine sweat gland neoplasm is important because of the potential for aggressive local growth and distant metastasis. FNAC plays an important role in the preoperative diagnosis and management of these lesions.

## Introduction

Aggressive digital papillary adenocarcinomas are rare eccrine sweat gland tumors which have the potential for highly aggressive biologic behavior. We report the cytological finding of a case of aggressive digital papillary adenocarcinoma. To the best of our knowledge, the cytology findings have not been reported in the English literature.

## Case Report

A 45-year-old female presented with a recurrent swelling over the lateral aspect on the dorsum of the right hand, of 5 months duration, associated with pain for the past 5 days. The local examination of the swelling revealed a 3×2 cm, tense, cystic, nontender swelling with restricted motility in the subcutaneous plane.

The general physical and systemic examinations were normal. No abnormality in the breast, thyroid, lungs, and abdomen was detected clinically. The routine laboratory investigations and chest radiographs and ultrasound scan of the abdomen were normal.

**Figure 1:**
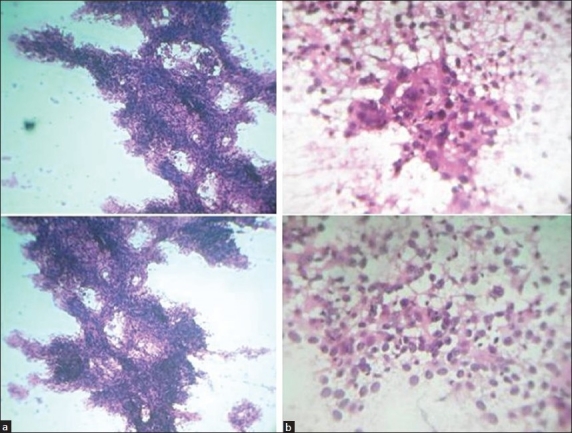
(a) Smears showing papillary tissue fragments of pleomorphic epithelial cells, traversed by fibrovascular core (H and E, ×100) (b) smears showing pleomorphic epithelial cells having scant cytoplasm, vesicular nucleus and prominent nucleoli (H and E, ×400)

Fine needle aspiration cytology (FNAC) of the swelling was done using a 24-gauge needle and 10-ml syringe and about 2 ml of blood mixed fluid was aspirated. Examination of hematoxylin and eosin (H and E) stained smears revealed highly cellular aspirates consisting of many tissue fragments of pleomorphic epithelial cells which were also scattered singly throughout the smears [Figure 1a]. The tissue fragments were traversed and surrounded by fibrovascular core [Figure 1a]. The cells had scant to moderate cytoplasm and a large vesicular nucleus and prominent nucleoli [Figure 1b]. A cytological diagnosis of malignant papillary epithelial cystic lesions suggestive of aggressive digital papillary adenocarcinoma was suggested.

**Figure 2:**
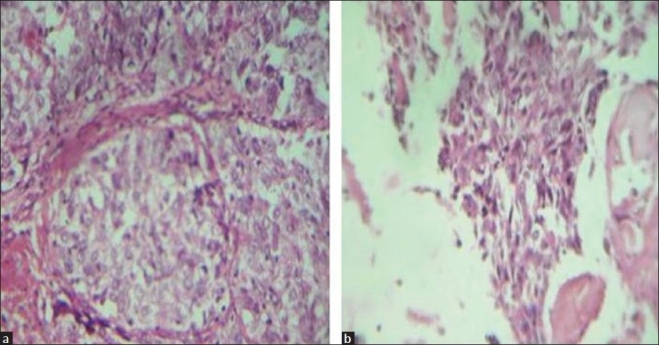
(a) Sections showing tubulo-alveolar structures occupying cystic lumina surrounded by fibrovascular core (H and E, ×400) (b) tumor cells are seen invading the bone (H and E, ×400)

An excision biopsy was done and subjected to histopathological examination. Grossly, the lesion was well circumscribed, measuring 2×1 cm. The cut section revealed solid and cystic areas. Microscopically, the lesion was characterized by tubulo-alveolar and ductular structures occupying cystic lumina. The stroma varied from thin fibrous septae to areas of dense hyalinized collagen. Fibrovascular core circumscribed the epithelial islands [Figure 2a]. Moderate cytological pleomorphism, mitotic figures and invasion into the bone were observed [Figure 2b]. A histological diagnosis of aggressive digital papillary adenocarcinoma, as suggested on cytology, was confirmed.

## Discussion

Aggressive digital papillary adenocarcinoma is a rare tumor arising from skin sweat glands. It occurs as a single painless mass, almost exclusively on fingers, toes and adjacent skin of palms and soles.[1–3] It occurs predominantly in men, with a mean age of 52 years.[1] It has also been reported in the younger age group of 15 years.[3,4] These tumors are multinodular, solid and cystic with papillary projections. The characteristic cytological features include tissue fragments consisting of syncytial aggregates and complex folded branching sheets of pleomorphic epithelial cells with a distinct anatomical border. The tissue fragments show nuclear crowding and overlapping. Fibrovascular core traverse or circumscribe the tissue fragments. The cells have enlarged, ovoid, pale nuclei, finely granular chromatin and distinct nucleoli with dense cytoplasm and distinct cell borders. The characteristic histological features include tubulo-alveolar and ductal structures with areas of papillary projections protruding into the cystic lumina. The stroma varies from thin, fibrous septae to areas of dense hyalinized collagen. The malignant features of the lesion are characterized by poor glandular differentiation, necrosis, cellular atypia and pleomorphism with invasion of soft tissue, bone and blood vessels.[1]

These tumors have a high rate of local recurrence.[1,5] In a study by Jih *et al*.,[5] the local recurrence in completely excised lesion was found to be 5%, and in untreated lesions it was 50%.

These tumors can metastasizse, occasionally resulting in mortality. It was found to be 4% in a study by Jih *et al*.[5] and 41.2% in a study by Kao *et al*.[1] Metastasis to the lung was commonly observed.[1,6] No evidence of metastasis was seen in the present case though features of local invasion were seen on histopathology.

These tumors may simulate metastatic papillary carcinoma from breast, lung, thyroid, ovary and lesion from these areas need to be ruled out by clinical and radiological examination. The other malignant skin adnexal lesions can be differentiated by the site of occurrence and non-papillary nature of these lesions.

## Conclusions

Aggressive digital papillary adenocarcinomas are rare, distinct, eccrine sweat gland malignant tumors characterized by aggressive biologic behavior with a high rate of local recurrence and distant metastasis, occasionally resulting in mortality. FNAC aids in establishing an early diagnosis and management of these cases.
